# The dysfunctional right ventricle: the importance of multi-modality imaging

**DOI:** 10.1093/ehjci/jeac037

**Published:** 2022-03-02

**Authors:** Elena Surkova, Bernard Cosyns, Bernhard Gerber, Alessia Gimelli, Andre La Gerche, Nina Ajmone Marsan

**Affiliations:** Cardiac Division, Department of Echocardiography, Royal Brompton Hospital, Part of Guy’s and St Thomas’ NHS Foundation Trust, London, UK; Department of Cardiology, Brussels University Hospital, Brussels, Belgium; Division of Cardiology, Department of Cardiovascular Diseases, Cliniques Universitaires St. Luc, Pôle de Recherche Cardiovasculaire (CARD), Institut de Recherche Expérimentale et Clinique (IREC), Université Catholique de Louvain, Av Hippocrate, 10/2806 Brussels, Belgium; Fondazione Toscana Gabriele Monasterio, Via Moruzzi, 1, Pisa 56124, Italy; Clinical Research Domain, Baker Heart and Diabetes Institute, Melbourne, Australia; Department of Cardiology, Leiden University Medical Centre, Abinusdreef 2, 2300RC Leiden, The Netherlands

**Keywords:** multi-modality imaging, right ventricle, myocardial function, arterial-ventricular coupling

## Abstract

Assessment of right ventricular (RV) function is crucial for the evaluation of the dyspnoeic patient and/or with systemic venous congestion and provides powerful prognostic insights. It can be performed using different imaging modalities including standard and advanced echocardiographic techniques, cardiac magnetic resonance imaging, computed tomography, and radionuclide techniques, which should be used in a complementary fashion. Each modality has strengths and weaknesses based on which the choice of their use and in which combination may vary according to the different clinical scenarios as will be detailed in this review. The conclusions from multiple studies using different imaging techniques are concordant: RV function can be reliably assessed and is a critical predictor of clinical outcomes.

## Graphical Abstract

**Graphical Abstract:**
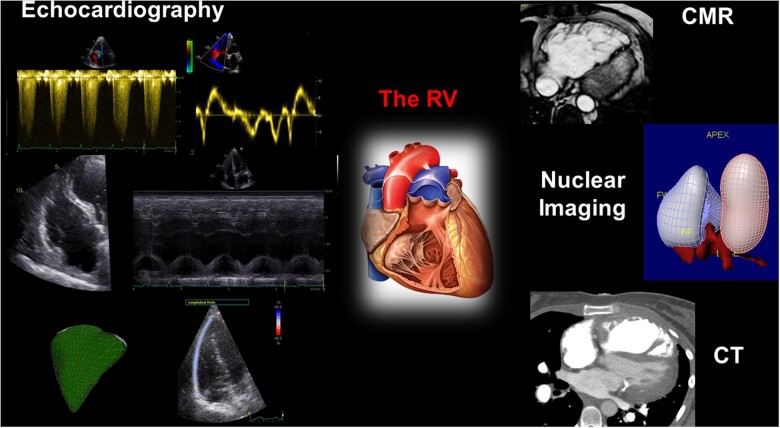
Multi-modality imaging plays a key role in the assessment of right ventricular (RV) morphology, size, and function.

## Introduction

The role of the heart is to provide sufficient cardiac output to meet the metabolic needs of the body at rest and during exercise. To achieve this, both the left ventricle (LV) and right ventricle (RV) must augment their function to meet demand. It may be said that ‘*you are only as good as your worst ventricle’* because the ventricles act in series as such that the output of one ventricle cannot exceed the other. Heart failure (HF) syndromes have been historically based on LV dysfunction as the source of limitation, and most therapeutic approaches aimed at improving LV remodelling and contractility. However, there are many settings in which an increased load of the RV and/or a decrease in RV contractility can result in a failure to appropriately augment cardiac output.

Excess loading and limitations in contractile force are therefore the key factors that may combine to limit RV output. Regarding RV load, it is critical to note that the RV sits ‘upstream’ of the systemic circulation, the LV, the left atrium, and pulmonary circulation. Dysfunction at any of these levels can result in increased pulmonary artery pressures and RV after-load, such as in HF with preserved or reduced ejection fraction (EF), valvular heart disease, and pulmonary vascular disease. With an RV mass and a contractile reserve of approximately one-third of the LV, RV output is highly sensitive to increases in after-load,^1^ and can decrease dramatically particularly during physical activity when increases in RV after-load, work and wall stress are far greater than for the LV.^2^ Thus, the concept of ventricular–arterial interaction is particularly important for the RV. Increase in RV after-load can be initially accommodated by an increase in RV contractility such that output is maintained. However, when sustained increases in loading cannot be matched by RV contractile reserve, ‘*uncoupling’* will occur as the RV is incapable of meeting the demands imposed on it. At first, the uncoupling presents during exercise or acute illness, but is then detectable at rest in more advanced disease stages.

These important physiological principles explain why assessment of the RV function is crucial for the evaluation of the dyspnoeic patient and/or with systemic venous congestion, and can provide powerful prognostic insights. RV function has been shown to be a critical determinant of survival in pulmonary vasculature diseases, such as pulmonary arterial hypertension,^3,4^ and also conditions that have traditionally been regarded as primarily LV pathologies such as congestive HF^5–7^ and acute myocardial infarction.^8–10^ The RV can be assessed using a range of imaging modalities including Doppler, 2D and 3D and strain echocardiographic techniques, magnetic resonance imaging (CMR), computed tomography (CT), and radionuclide techniques. Recent technological advances have improved the feasibility and accuracy of each imaging modality for the assessment of RV function, but still there is no single approach able to provide all the necessary information, and their use in a complementary fashion is therefore advised (*Graphical Abstract*). As summarized in *Table 1* and will be detailed in this review, each modality has strengths and weaknesses, based on which the choice of their use and in which combination may vary according to the different clinical scenarios. The conclusions from multiple studies using different imaging techniques are concordant: RV function can be reliably assessed and is a critical predictor of clinical outcomes.

**Table 1:** Strengths, limitations, and value in the clinical setting of each imaging modality for the assessment of RV function

Parameter	Strengths	Limitations	Use/value in the clinical setting
Echocardiography			
TAPSE	Easy, fast, and widely available Reproducible Established prognostic value	Reflects only longitudinal function Neglects contribution of IVS and RVOT Uses extracardiac reference point Angle- and load-dependent	Useful for the first assessment (at bed side) and in emergency settings Not advised in post-cardiac surgery patients Needs to be always combined with other measures of RV function
*S*′ (TDI)	Easy and fast Reproducible Established prognostic value	Reflects only longitudinal function of basal lateral segment Neglects contribution of IVS and RVOT Angle- and load-dependent	Not advised in post-cardiac surgery patients Needs to be always combined with other measures of RV function
FAC	Easy and widely available Reflects both longitudinal and radial contraction Fair correlation with CMR-derived RV EF Established prognostic value	Heavily dependent on image quality Poor reproducibility Neglects contribution of the RVOT Load-dependent	Should be used in addition to parameters reflecting longitudinal function, ideally in patients with good endocardial border delineation Contrast should be considered if poor acoustic window
RIMP (TDI)	Provides information on global RV function Less dependent on acoustic window	Unreliable when RA pressures are elevated Limited use in normal RV	Could be used as a part of multi-parametric approach, especially in patients with suboptimal image quality, when other parameters are less reliable
*dp*/*dt*	Easy and widely available	Inapplicable in patients with no/trivial TR or severe TR Load-dependent	Not widely recommended for routine clinical use
2DE longitudinal strain	Measures longitudinal myocardial deformation Less angle- and load-dependent Less confounded by RV geometry and passive motion Reproducible Established prognostic value, additive to other RV parameters	Requires good image quality Neglects contribution of the RVOT Requires post-processing Vendor-dependency, for which caution is required in follow-up studies Relatively limited availability	Should be used routinely for dedicated RV assessment, especially in subgroups of patients with RV involvement Important prognostic marker, superior to other conventional RV parameters Allows to diagnose subclinical RV dysfunction Mechanical dispersion index can be used as a measure of RV dyssynchrony
3DE EF	Independent of geometric assumptions Extensively validated against CMR Established prognostic value, superior to other RV parameters	Heavily dependent on image quality Need for patient’s co-operation (± regular R–R intervals in case of multi-beat acquisition) Limited availability Requires specific equipment and dedicated analysis software Requires post-processing Requires specific training	In echocardiographic laboratories with good expertise in 3Dechocardiography, 3D-derived RV EF should be used routinely for assessment of RV systolic function in patients with good acoustic window
3D shape	Provides additive prognostic information in specific patients’ populations	Not yet available for routine clinical use Requires further post-processing of 3D full-volume datasets and all limitations of 3DE apply	Currently for research use mainly
RV–PA coupling	Reflects both RV after-load and contractility Established prognostic value	Inapplicable in patients with no/suboptimal TR Doppler signal Complexity and availability depend on the parameter used for assessment of RV contractility (TAPSE, FAC, strain, 3D RV EF), with their limitations apply	Can be used in addition to other parameters of RV function for dedicated RV assessment, especially in subgroups of patients with RV involvement
Myocardial work	Reflects RV after-load, contractility, and dyssynchrony	Inapplicable in patients with no/suboptimal TR Doppler signal All limitations of 2DE longitudinal strain apply	Can be used in addition to other parameters of RV function for dedicated RV assessment (and mainly in follow-up studies), however data on clinical/prognostic value are still limited
CMR			
EF	Gold-standard imaging modality for assessment for RV volumes and EF Excellent image quality/endocardial borders delineation in majority of patients Free from acoustic window limitations Independent of geometric assumptions Established prognostic value	Costly and time-consuming Limited availability Limited use/suboptimal quality in patients with arrhythmias or poor breath holding Limited use in claustrophobic patients Impaired image quality/accuracy in patients with intracardiac leads Challenging positioning of basal slice during post-processing Requires experience Use limited to clinically stable patients	Should be used for dedicated assessment of the RV volumes and EF, especially if RV myocardial tissue characterization is required (e.g. presence of fat, fibrosis) or/and if change in management plan may follow (e.g. selection for surgery) or anatomical details are necessary (congenital heart disease) Can be combined with measures of longitudinal function (TAPSE, feature tracking strain)
Nuclear imaging			
EF	Independent of geometric assumptions Free from acoustic window limitations Extensively validated	Costly and time-consuming Use of radiation Low temporal resolution Use limited to clinically stable patients	Measures of RV function can be combined with perfusion and metabolism assessment (e.g. in suspected RV involvement or infarction or in pulmonary hypertension)
CT			
EF	Independent of geometric assumptions Free from acoustic window limitations	Costly Use of radiation Use of contrast Requires stable cardiac rhythm with relatively low heart rate Low temporal resolution Use limited to clinically stable patients	Can be used in patients undergoing CT for other clinical indications (e.g. assessment of pulmonary venous drainage, valve percutaneous interventions etc.)

CMR, cardiac magnetic resonance imaging; CT, computed tomography; EF, ejection fraction; FAC, fractional area change; IVS, interventricular septum; PA, pulmonary artery; RA, right atrium; RVOT, right ventricular outflow tract; TAPSE, tricuspid annular plane systolic excursion; TDI, Tissue Doppler imaging; RIMP, RV index of myocardial performance.

## RV assessment by echocardiography

### 2D echocardiography

In daily clinical practice, RV evaluation is mainly performed by 2D echocardiography. However, there is currently little uniformity in echocardiographic approach to the RV, owing to potential lack of awareness and/or conformity with recommendations.^11^

Qualitative evaluation of the RV anatomy should be performed from multiple acoustic windows and is particularly important in the context of ischaemic heart disease or arrhythmogenic right ventricular cardiomyopathy (ARVC, for detection of wall motion abnormalities), and suspicion of constriction or pressure/volume overload (assessment of septal motion patterns).

The quantitative assessment of the RV size should include several measurements. RV-free wall thickness can be measured at end-diastole in the subcostal four-chamber view or parasternal view: >5 mm is considered abnormal after exclusion of the trabeculae. RV basal, mid-cavitary diameters, and base-to-apex length are also recommended, together with the RV outflow tract (RVOT) diameter (measured above aortic valve) in the parasternal long-axis view, as well as RVOT proximal and distal diameters in the parasternal short-axis view.^12^ These measurements are particularly important as part of the criteria for the diagnosis of ARVC. However, the operator should ensure a correct alignment of the views to provide accurate and reproducible values. RV end-systolic area (ESA) and end-diastolic area (EDA) are obtained by manually tracing the ventricular endocardium in the RV-focused apical four-chamber view, excluding the trabeculae: normal values can be found in the ASE/EACVI recommendation paper.^12^

The quantitative evaluation of RV performance is quite challenging and can be estimated with different parameters. Fractional area change (FAC) is calculated by the formula [(EDA − ESA)/EDA] × 100, and showed fair correlation with RVEF assessed by CMR: a value <35% is considered abnormal.^13^ The ability of FAC to predict major adverse cardiac events has been demonstrated in various cardiovascular conditions including myocardial infarction and pulmonary hypertension.^9,14^ 2D RVEF is not recommended for clinical use due to the inaccuracy of the method.^12^ The tricuspid annular plane systolic excursion (TAPSE) quantifies the excursion of the lateral tricuspid annulus from end-diastole to end-systole and reflects only the longitudinal contraction of the RV-free wall. This simple and reproducible measure is considered abnormal when <17 mm. Many assumptions and drawbacks should be considered while using this parameter (angle- and load-dependent, inaccurate in patients post-cardiac surgery or in case of RV pacing/RV apex rotation, etc.). Nevertheless, its prognostic value has been demonstrated in several conditions such as HF, coronary artery disease, pulmonary embolism, pulmonary hypertension, and in critically ill patients.^15,16^ Tissue Doppler imaging (TDI)-derived tricuspid lateral annular systolic velocity (*S*′) is another parameter of RV longitudinal function, which correlates with CMR-derived RVEF^13^: a value <10 cm/s is considered abnormal but may decrease with age. The limitations are similar to those for TAPSE and should be taken into account. RV index of myocardial performance (RIMP) is a ratio between isovolumic contraction and relaxation times and the ejection time. This parameter reflects global RV systolic function and was shown to correlate well with CMR-derived RVEF. RIMP <0.55 is considered abnormal. Another measure of RV function is the RV *dp*/*dt*, which represents the rate of change of pressure and is calculated as the slope of the spectrum of tricuspid regurgitation between 1 and 2 m/s: a value <400 is considered abnormal. This parameter is less used due to its high load dependency. Finally, a less load dependent parameter of RV performance is the right isovolumic myocardial acceleration (IVA), which is the peak isovolumic myocardial velocity divided by the time to reach this peak velocity: it is considered abnormal when <2.2 m/s.

Assessment of RV systolic function during exercise stress echocardiography is a promising diagnostic and prognostic tool. Exercise-induced reduction in TAPSE demonstrated important incremental prognostic value in asymptomatic patients with primary mitral regurgitation.^17^ Attenuated increase in FAC and *S*′ with exercise indicated subclinical RV dysfunction in athletes with RV arrhythmias.^18^

2D speckle tracking echocardiography (STE) has recently emerged, enabling a real-time tracking of the frame-to-frame myocardial motion, and overcoming most of the limitations inherent in conventional echocardiography. In particular, STE is less angle- and load-dependent, and less influenced by passive tethering, thus allowing accurate quantification of regional and global myocardial function, reflecting more closely myocardial contractility.^19^ RV peak systolic longitudinal strain and strain rate are assessed in the (RV-focused) apical four-chamber view, where RV-free wall and septum are each divided into three segments (basal, middle, and apical). Regional strain values are generated, and the RV four-chamber longitudinal strain (average of the six segments) and RV-free wall strain (average of the three segments) are derived.^19^ Current recommendations propose a cut-off value of −20% for RV four-chamber longitudinal strain and of −23% for RV-free wall strain as abnormal. However, some studies showed that a lower cut-off for RV four-chamber strain of −19% was associated with poor prognosis. Overall, RV longitudinal strain has shown important prognostic value in various conditions, including pulmonary hypertension, valvular heart disease, LV dysfunction, and HF.^20^ Recent studies suggest that the mechanical dispersion of the strain curves could also play a role in the risk stratification of patients with ARVC.^21^

**Figure 1:**
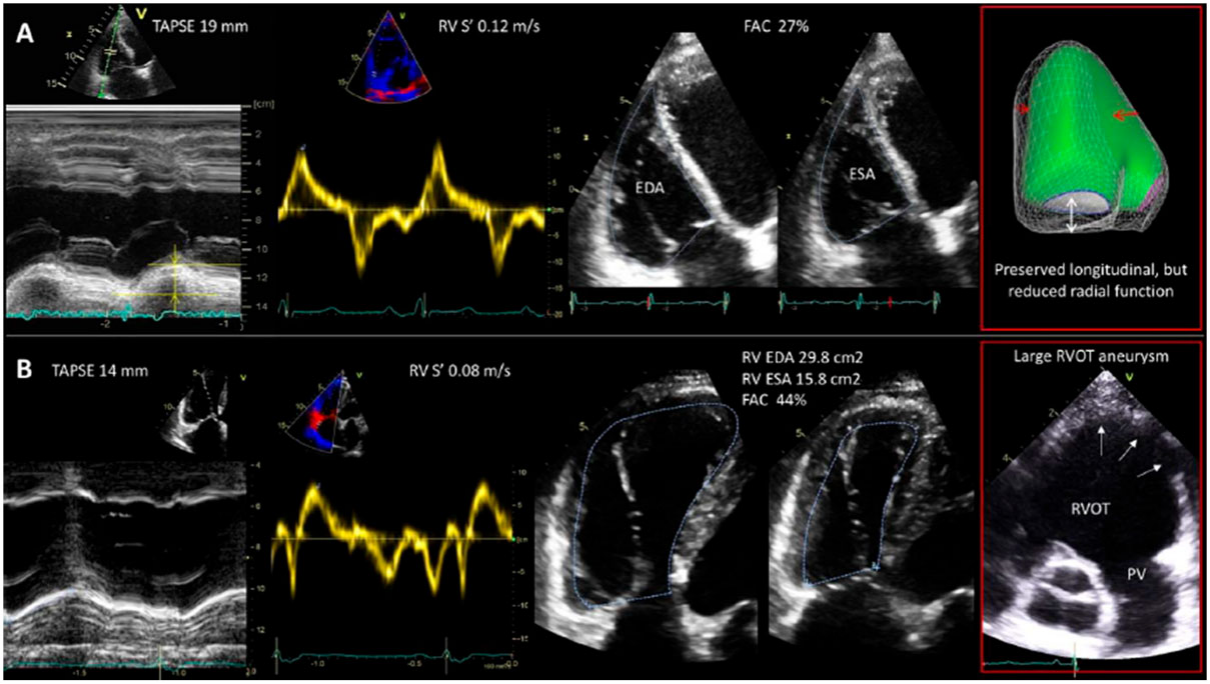
Importance of multi-parametric approach in accurate interpretation of RV systolic function by conventional echocardiography. (*A*) In a patient with pulmonary hypertension, TAPSE, and *S*′ values are normal, however FAC is reduced. 3D surface-rendered model of the RV demonstrates preserved longitudinal contraction (white arrow), but impaired radial contraction (red arrows) due to reduced RV-free wall motion and flattening of the interventricular septum. FAC considers not only longitudinal but also radial RV contraction and in this case is able to reflect the reduced RV systolic function. (*B*) In a patient with repaired Tetralogy of Fallot, TAPSE, and *S*′ are reduced (common post-cardiac surgery), but FAC is normal. 3D-derived RV EF is reduced (40%). This is explained by large dyskinetic area in the RVOT (last panel, arrows) which was not included neither in TAPSE nor in *S*′ or FAC measurements.

Importantly, assessment of the RV size and systolic function by conventional echocardiography should always be performed in a clinical context, including RV loading conditions. Accurate evaluation requires multi-parametric approach and use of different echocardiographic views to ensure correct interpretation of the findings, especially when there is discrepancy between echocardiographic parameters, as illustrated in *Figure 1*.

### 3D echocardiography

**Figure 2:**
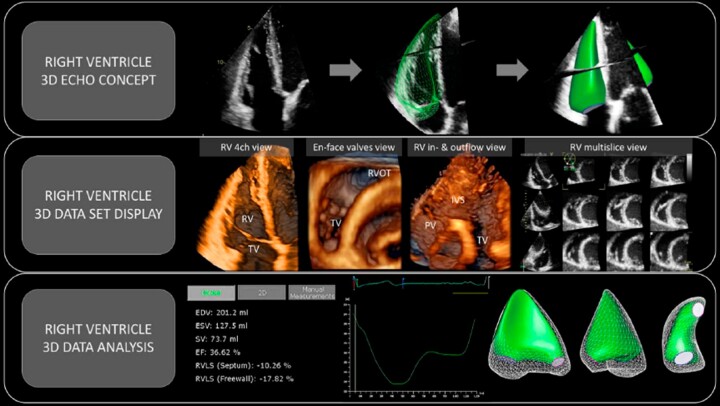
3D echocardiographic assessment of the RV. (*Upper*) Standard 2D echocardiography cannot consider contribution of the RV parts not captured in the scanning plane. 3D echocardiography allows to incorporate all RV components in a single dataset (green surface-rendered model) and enables accurate assessment. (*Middle*) The 3D full-volume dataset of the RV can be cropped, rotated, and displayed in 3D volume rendering mode or multi-slice display, and provide information targeted to exam question (e.g. assessment of RV morphology, valves, RVOT, or interventricular septum). The multi-slice view is useful during data acquisition to ensure that the whole RV is encompassed in the dataset and can also be used for assessment of RV wall motion abnormalities. (*Lower*) Post-processing using dedicated software packages provides RV volumes and EF. It also generates the volume curve which details emptying and filling of the RV during cardiac cycle. Different views of surface-rendered 3D model of the RV enable visual assessment of the RV dynamics and contribution of different components of the RV pump function.

Unlike 2D echocardiography, 3D echocardiography enables acquisition of full-volume datasets from either a single beat capture or few consecutive beats with sub-volumes stitched together, allowing for higher temporal and spatial resolution. This full-volume dataset incorporates all three components of the RV (inflow, apical portion, and outflow) and allows for a detailed analysis of RV size, shape, function, and contraction patterns (*Figure 2*). In particular, RV full-volume dataset can be post-processed using dedicated software packages to obtain tracing of the RV endocardial surface at end-diastole and end-systole and measurement of RV volumes and EF, without using any geometrical assumptions or formulas.

RV volumes and EF derived from 3D echocardiography are known to correlate well with CMR data both in children and adults.^22,23^ High accuracy and reliability of 3D echocardiography has also been confirmed in a meta-analysis comparing the performance of different imaging modalities using CMR as reference method: 3D echocardiography was observed to slightly overestimate RVEF^24^ and underestimate RV volumes.^23^ Thus, CMR and 3D echo values and reference ranges cannot be used interchangeably. Large 3D echocardiography studies conducted in healthy subjects have provided sex-, age-, and body size-specific reference values for RV volumes and EF.^25^ Importantly, 3D echocardiographic assessment of RV volumes and EF is recommended by current guidelines as a method of choice and a RVEF >45% is considered as the lower limit of normal.^12^ It is important to note that accurate 3D echocardiographic quantification of RV volumes and EF requires experienced personnel and the fact that only 12% of European centres use 3D assessment of RV systolic function on a regular basis highlights the need for further training.^26^ Principal technical limitations of 3D echocardiography include dependency on image quality and potential incomplete visualization of the whole cardiac chamber in case of severe RV dilation. Multi-beat acquisition also relies on regular heart rate and patients’ co-operation with breath holding. However, 3D echocardiography offers multiple operational and safety benefits including its wide availability, portability, absence of ionizing radiation, and safe examination of patients with intracardiac devices. Recent improvements in echo techniques and data analysis, such as single-beat 3D data acquisition with high volume rate, advanced software solutions for the volumetric analysis of the RV on board of the echocardiographic scanners, semiautomated algorithms allowing for fast, and reproducible volumetric analysis, have defined the 3D echocardiography as an established and versatile technique for the assessment of the RV performance.

Regarding its prognostic value, 3D echocardiography-derived RVEF has been shown to be independently associated with cardiac and all-cause mortality and major adverse cardiac events (MACE) in patients with various cardiovascular conditions.^27,28^ RVEF offers improvements in prediction of adverse clinical outcomes compared with other parameters including LV systolic and diastolic function or clinical risk factors. Partition values of the RVEF for mild (EF = 40–45%), moderate (30–40%), or severe (<30%) RV dysfunction have been also prognostically validated to stratify the risk of cardiac death and MACE.^27^ Prognostic value of RVEF was shown to be superior to conventional echocardiographic parameters of RV systolic function for predicting mortality.^28^

Quantification of the individual motion components of the RV using 3D echocardiography enables a comprehensive characterization of RV contraction pattern, which could be abnormal even in the context of normal RVEF.^28,29^ Recently developed software package enables to decompose the wall motion of the 3DE-derived RV model along three anatomical axes: longitudinal (from tricuspid annulus to the apex), radial (perpendicular to the interventricular septum), and anteroposterior (parallel to the interventricular septum) axes and quantify contribution of each motion component separately.^30^ The inward motion of the free wall (radial shortening) and interestingly, the anteroposterior shortening (mainly caused by the stretching of the RV-free wall insertion points during the LV contraction) was shown to be as important as longitudinal shortening in healthy volunteers.^31^ Also, RV pressure overload could be detected early if radial motion component was lost, while RV volume overload showed more prominent impact on the longitudinal RV component.^32^ However, data on the prognostic role of different components of RV systolic function remain scarce. A recent study on a large cohort of patients with left-sided heart disease demonstrated that in a subgroup of patients with preserved RVEF, reduced relative contribution of the anteroposterior component was significantly and independently associated with adverse outcomes, while RVEF or LVEF was not.^29^

Finally, the role of RV remodelling and RV shape alterations are widely recognized in RV pressure and volume overload conditions. However, till recently there was no evidence supporting association of poor outcomes in patients with adverse RV shape changes independent of those predicted by size and functional parameters. A unique approach by 3D echocardiography to provide RV shape indices based on analysis of the RV curvature was recently developed and evaluated in normal subjects and in patients with pulmonary arterial hypertension.^33^ In patients with pressure overload, the curvature of the RV inflow tract was a more robust predictor of death than RVEF, RV volumes, or other regional curvature indices. Recently the reference values of 3D echocardiographic values of regional curvature indices were reported in a large group of healthy subjects to facilitate future studies on adverse RV remodelling.^33,34^

### Right ventricular arterial coupling and myocardial work

**Figure 3:**
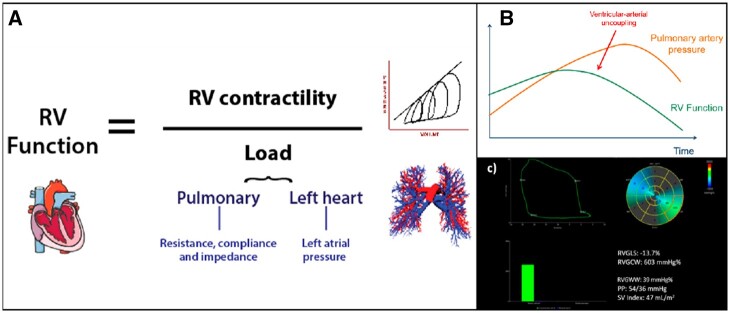
Right ventricular—arterial interaction. RV function, when measured by tools such as ejection fraction, area change, and strain, is the result of the interaction between the intrinsic muscular force of the RV (contractility) and after-load (often simplified as pulmonary artery pressure)—see panel *A*. These measures therefore do not enable us to determine whether the RV is matching its work requirements relative to load or whether it is approaching the point of uncoupling (*B*) at which cardiac output starts to fail relative to demand. RV myocardial work (MW) can be calculated based on non-invasively derived pressure–strain loops for the RV, in this case in a patient with raised pulmonary arterial pressures (*C*). RVGCW, right ventricular global constructive work; RVGWW, right ventricular global wasted work.

Many conventional measures of RV function, such as FAC, EF, and TAPSE, reflect the interaction between loading conditions and contractility. However, an accurate measure of (load-independent) contractility represents the ideal means of determining the patient’s prognosis and possibly the need for intervention. A load-independent RV function assessment can theoretically be achieved by two means—either a direct load-independent surrogate of contractility or by incorporating load into the measurement of RV function (*Figure 3*).

#### Load-independent RV function measures

Only few measures have been proposed as surrogate approximates of RV contractility. Two decades ago, Vogel *et al.*^35^ demonstrated a close relationship between IVA and invasively measures of contractility (preload recruitable stroke work and *dP*/*dT*max) under a range of varied loading conditions. The theory was intriguing given that IVA is a measure of myocardial motion during the isovolumic contraction period, prior to the ventricle being exposed to its after-load. Whilst the concept is appealing, the measure has not been well validated or widely accepted, perhaps due to difficulties with reproducibility. As mentioned above, STE is another approach promoted as being an approximate of contractility: longitudinal strain is a measure of deformation and, both in theory and in practice, should be interpreted relative to load. However, based on animal and human studies,^36^ the change in strain relative to time (strain rate) might be a closer approximate of contractility. There is some support for this premise, but strain rate has also failed to make its way into clinical routine due to issues with reproducibility and its impact on a measure with small dynamic range.

#### Load-adjusted RV function measures

An alternate means of dissecting the underlying RV contractility is to express it as the relationship between RV function and load. This is akin to the approach in which contractility can be determined invasively from pressure volume–volume relationships. In a series of elegant studies, Guazzi *et al.*^37^ have demonstrated the prognostic utility of expressing TAPSE relative to pulmonary artery systolic pressure (PASP) estimates as a surrogate of contractility. A variant on this concept incorporates the ratio of RV area to PASP and has been validated against a gold-standard hybrid using CMR volumes and invasive pressure measures, both at rest and throughout exercise.^38^ The utility of these measures during exercise provides an additional strength in that the additional load and work of exercise challenges the contractile reserve of the ventricle. In essence, this provides an insight into whether the compensated RV is starting to fail.

#### Right ventricular myocardial work

Recently, a non-invasive method to assess LV myocardial work (MW has been proposed incorporating STE-derived LV global longitudinal strain and non-invasive brachial cuff blood pressure measurements. This approach has been validated against pressure–volume loops derived invasively and showed its value in different patient populations as a comprehensive measure of LV function, which considers both LV after-load and the possible presence of post-systolic shortening/dyssynchrony.^39^ The same method can be applied for the RV, using the pulmonary pressures as an estimate of myocardial force, and STE-derived RV longitudinal strain to estimate changes in segment length (*Figure 3*). RV MW therefore provides an integrative analysis of RV function incorporating RV longitudinal strain with pulmonary pressures, as measure of RV after-load, although an accurate estimation of the last one can be challenging in some cases. Cardiac cycle timings are determined by pulmonic and tricuspid valve opening and closure events, identified through either direct visualization of 2D images or by pulsed-wave Doppler interrogation. Any myocardial lengthening occurring during systole and any shortening during isovolumic relaxation is recorded as RV wasted work (RVGWW, mmHg%); therefore, any inefficient post-systolic shortening will not contribute to estimate the RV constructive work (RVGCW, mmHg%), defined as the work contributing to the shortening of the cardiac myocytes during systole and the lengthening during isovolumic relaxation. A recent study showed a significant correlation of these novel indices derived non-invasively by pressure–strain loops, and in particular RVGCW, with invasively measured stroke volume index.^40^ The importance of considering after-load in the assessment of RV function has been demonstrated by studies on RV–pulmonary arterial coupling.^37^ This is particularly relevant even when using RV longitudinal strain, which remains after-load-dependent even more than LV longitudinal strain due to the thinner walls and lower ventricular elastance of the RV. Furthermore, RVMW synchronizes pulmonic and tricuspid valvular events with RV longitudinal strain, accounting also for possible post-systolic shortening (often seen in the setting of pulmonary hypertension) and septal dyssynchrony due to ventricular interdependence.^41^ RVMW indices have the potential of delivering a more precise estimate of RV systolic function and may enhance the non-invasive understanding of the pathophysiology of patients with HF, pulmonary hypertension, and any other form of RV involvement, also providing a new tool to non-invasively characterize their response to therapies. Further validation of this approach (currently also based on a single-vendor software) is necessary to support its application in the clinical practice and studies on its potential prognostic value are warranted.

## RV assessment by CMR

CMR is currently considered the gold standard for assessment of RV function and structure. It allows indeed high-resolution time-resolved 3D visualization of the complex anatomical shape of the RV without most of the limitations that hinder other imaging modalities such as limited acoustic windows, body size or deformation, and anatomical changes of the RV due to pathology or surgery. The standard technique for imaging volumetric and functional data of the RV is currently 2D steady-state-free precession (SSFP) cine imaging with retrospective ECG gating allowing to fully cover the RV with 10–12 slices acquired in 5–12 short breath-holds. Alternatively, in patients unable to breath-hold or in patients with multiple arrhythmias, images can also be acquired in free-breathing real-time images, but with less spatial and temporal resolution. Recently, using parallel imaging techniques or under-sampling with iterative reconstruction it is also possible to acquire true 3D cine breath-hold imaging.

**Figure 4:**
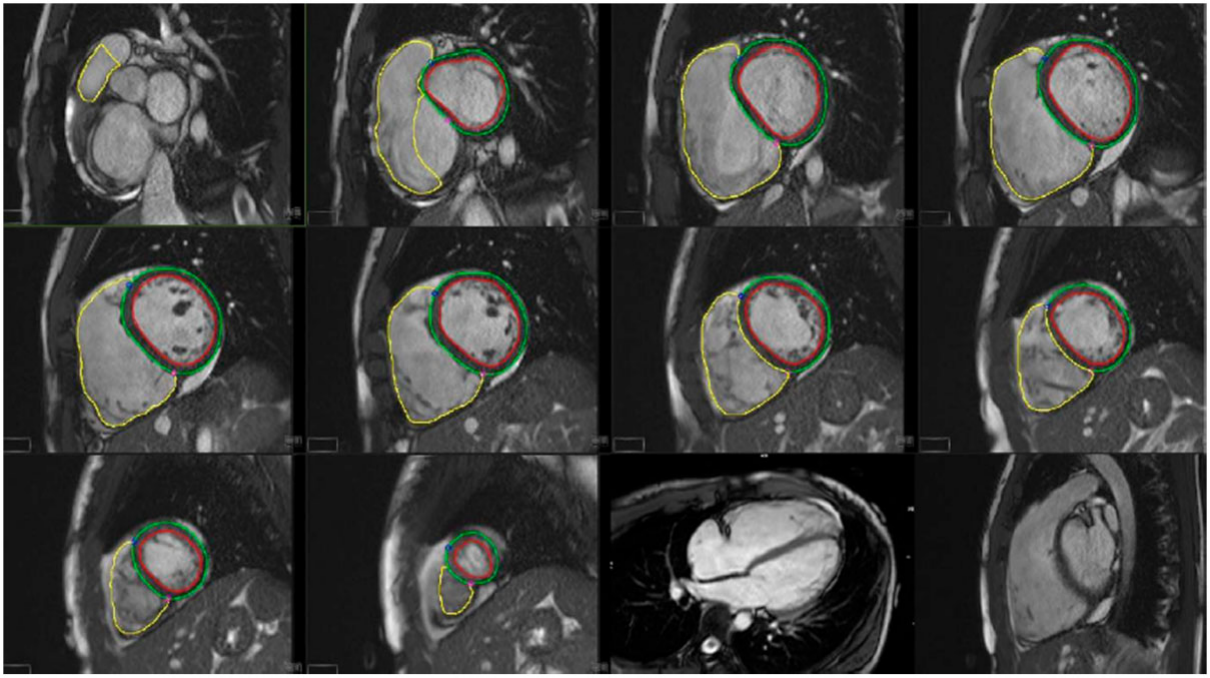
Volumetric assessment of RV function by CMR. Traces of the LV endocardium (red) epicardium (green) and the RV endocardium (yellow) are contoured in a short-axis stack with reference to the long-axis plane. Volumes are then imputed using a sum-of-disks method. In this case, an RV ejection fraction of 42% was measured in a young athlete presenting with symptomatic ventricular tachycardia, focal regions of RV akinesia, and widespread T-wave inversion meeting task-force criteria for ARVC.

The most commonly used approach for measurement of both RV and LV volumes is to acquire serial slices in short-axis view, covering both ventricles from apex to base (*Figure 4*). Imaging in axial plane of multiple axially rotated long-axis slices may allow better identification of the tricuspid valve plane and more precise evaluation of RV volumes and EF than imaging in short-axis plane especially in congenital heart diseases. However, this requires separate acquisitions for the RV and lengthens the exam duration.

For estimation of RV end-diastolic and end-systolic volumes and RVEF, the endocardial borders of both the LV and RV are traced in end-diastole and end-systole; and end-diastolic and systolic volumes are computed using Simpsons method by summing areas of traced cavity volume times slice thickness. Tracing can be performed manually or automatically. Recently deep-learning methods have been also proposed allowing automatic contour detection of the RV. To measure RV mass, epicardial contouring needs also to be performed. Epicardial RV volume is then subtracted from endocardial contours and multiplied by density of 1.06 to yield mass.

Because of the excellent differentiation of blood from RV tissue, the method is highly accurate. The main source of error arises from differentiating RV from right atrial volume in the most basal slice, which is somewhat more difficult for the RV than for the LV, because of the thinner RV-free wall and the larger excursion of the tricuspid annular plane as compared with the mitral one. The normal values for RV volumes, mass, and EF and their interpretation have been established by a multitude of studies in large populations such as the MESA study, the Framingham population, or the UK Biobank both in adults and children, and for non-Caucasian populations^42–44^ RV volumes and mass depend on body size and are thus generally indexed to body surface area. Indexed RV volumes and mass are generally greater in men than in women, depend on race and decrease with increasing age. Higher amounts of physical exercise are associated with larger RV mass and volumes, however unchanged RVEF. Obesity by opposition is also associated with an increase in BSA-indexed RV ESV and mass. By contrast to volumes, RVEF is less influenced by age, but is generally greater in women than in men.^44,45^ In children, an exponential relation between body surface area and RV volumes and mass was found and therefore reported in percentiles and/or *z*-scores.

Longitudinal RV function can also be evaluated by estimation of TAPSE or by RV feature tracking strain imaging computed on four-chamber cine imaging. Other approaches for computation of RV strains require specific sequences such as DENSE or FastHARP, which have the advantage of higher spatial resolution. However, tagging is less useful for the RV strain computation because of the thin RV wall relative to tag spacing (6–7 mm), allowing only computation of longitudinal but not of circumferential or radial RV strains.

CMR radiomics is also a novel technique for advanced image phenotyping based on the analysis of multiple quantifiers of shape and tissue texture, which has been applied also to the RV.^46^ Its application in the clinical practice is still limited due to reproducibility issues, for which machine learning models are being currently applied to improve the performance.

Finally, CMR also has the unique advantage of allowing RV myocardial tissue characterization. Fat infiltration in the RV, which is of particular interest in diagnosis or ARVC can be identified by T1 weighted black-blood spin echo imaging and confirmed by fat saturation techniques. RV fibrosis and necrosis may be present in a multitude of RV diseases and can be identified by late-gadolinium imaging (LGE) after injection of contrast. Also, it was also recently shown that after injection of contrast agents CMR also allows for estimation of pulmonary transit time and blood volume,^45^ allowing for assessment of pulmonary congestion in various situations of RV pressure overload.

### Clinical applications

The evaluation of RV function and structure by CMR has shown clinical relevance in multiple diseases, and particularly in congenital heart diseases, in ARVC, in pulmonary hypertension, in ischaemic diseases, and HF.

CMR plays a particular role in determining the aetiology of RV dilatation. An example is the diagnosis and evaluation of severity of atrial septal defects. Also, by assessing global and regional RV dilatation and by revealing structural abnormalities such as fat infiltration and RV fibrosis CMR may confirm diagnosis of ARVC. The current task force criteria rely on CMR volume and EF measurement to define major and minor criterial for ARVC.^47^ The identification of fat or LGE is not currently part of ARVC diagnosis but may be particularly useful in orientating the diagnosis. Two studies demonstrated that presence of such anomalies had additional prognostic value over ECG findings in ARVC patients or mutation carriers.^48^

CMR also plays a particular important role in various congenital heart diseases, from patients with either congenitally corrected transposition of RV or which have undergone atrial (Mustard or Senning) switch repair surgery to patients with complex congenital heart disease such as functional univentricular hearts with various palliation surgeries. In this setting the evaluation of RV fibrosis by LGE imaging has also been shown to provide prognostic value. CMR also plays a fundamental role in the follow-up of patients with tetralogy of Fallot (TOF). Indeed, relief of RVOT obstruction in TOF often involves disruption of pulmonary valve integrity, which leads to residual pulmonary regurgitation of various degrees in most patients. While this condition is often well tolerated, when severe pulmonary regurgitation is longstanding, it leads to progressive dilatation of the RV, causing RV failure and arrhythmias. Because of its high precision, and non-invasiveness CMR is ideally suited for longitudinal follow-up in patients with repaired TOF. It was shown that moderate or severe LV or RV systolic dysfunction by CMR, but not pulmonary regurgitation fraction or RV diastolic dimensions, is independently associated with impaired clinical status in long-term survivors of TOF repair.^49^ Also, the repair of pulmonary regurgitation in tetralogy of Fallot was shown to improve RV dilatation and clinical status. Although patients improve RV volumes after repair of pulmonary regurgitation, normalization was achieved only when dilatation was not excessive, with indexed RV end-diastolic volumes > 150–170 mL/m^2^ and systolic volumes >80–90 m/m^2^.^49^ Therefore, CMR is currently recommended in all patients with TOF to serially follow RV volumes and eventually provide timely indication for surgery.

Moreover, CMR also plays an important role Ebsteins’ anomaly. In this condition, which is characterized by severe tricuspid regurgitation due to anterior displacement of the septal leaflet of the tricuspid valve, it is particularly well suited to precisely distinguish the functional RV from the atrialized part of the RV and to measure their respective sizes.^50^ Similarly in other settings of tricuspid regurgitation, CMR also allows precise evaluation of RV dilatation and systolic function, which is of fundamental role to give surgery indication.

CMR was recently shown to play an important role in pulmonary hypertension. In this setting several studies have shown that RV function assessment by CMR predicts outcome and allows to evaluate response to therapy.^51^ Similarly, several recent studies have also highlighted the role of CMR–RV function on outcome of cardiomyopathies and ischaemic heart disease (including RV infarction), as well as in both HF with preserved or reduced EF.^6,52^

## RV assessment by nuclear imaging

Decades ago, radionuclide techniques have been the first imaging modalities used to provide accurate and reproducible measurements of RV structure and function. Nowadays, nuclear imaging still plays a role in selected subgroup of patients, being able to assess RV perfusion and metabolism as well as morphology and EF, and therefore providing new opportunities for comprehensive evaluation of RV from a single study.

**Figure 5:**
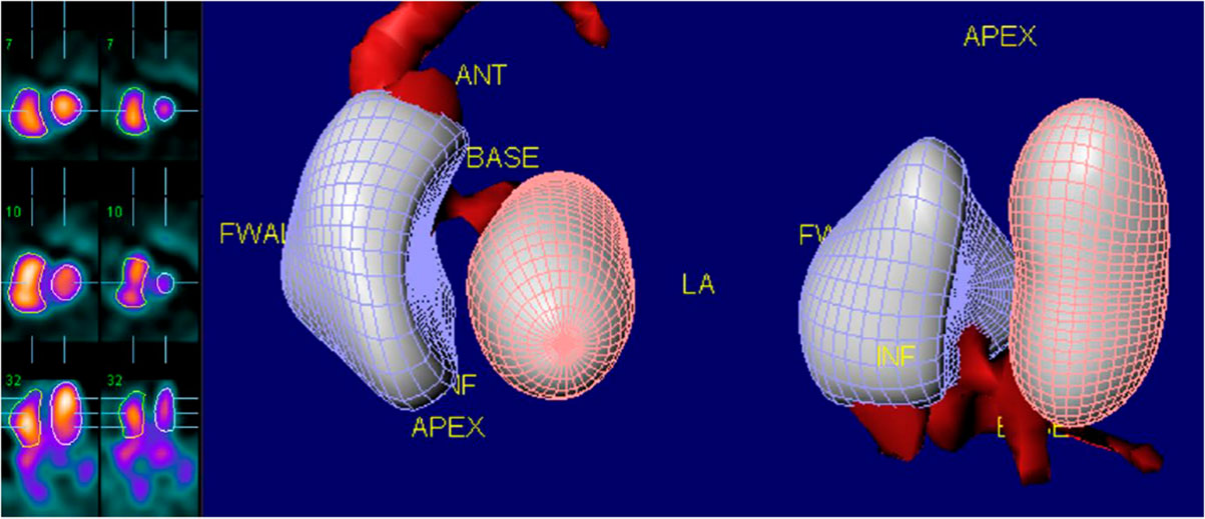
Example of SPECT radionuclide angiography. Regional and global evaluation of kinesis as well as quantitative evaluation of right and left ventricular ejection fraction and volumes are feasible and reproducible.

Assessment of RVEF using radionuclide techniques can be performed with either first pass (FPRNA) or equilibrium techniques (planar ERNA, SPECT ERNA), in which the radioisotopes in the blood pool can be imaged. Both these approaches have been extensively validated and, since RVEF is derived from end-systolic and end-diastolic count densities, it is independent of the geometric assumption required for other modalities.^53^ SPECT equilibrium radionuclide angiography, due to its 3D view, improves spatial separation and resolution of cardiac chambers and has been shown to provide accurate and reproducible assessment of RV volumes and EF (*Figure 5*).^53^

Nuclear-imaging techniques are emerging as clinically useful tools to assess RV perfusion and metabolism, to detect isolated RV infarction of RV or LV infarction with RV involvement. To improve visualization of RV, it’s possible to use a dedicated computed post-processing technique that can mask activity of LV. Previous studies showed that SPECT provides accurate detection of RV ischaemia in patients with suspected coronary artery disease, identified by reversible defects in the RV and interventricular septum and decreased RVEF during stress. Furthermore, PET allows non-invasive quantification of regional myocardial blood flow and coronary flow reserve in RV mostly using nitrogen-13 ammonia.

Finally, the evaluation of shift in fatty acid metabolism, assessed by SPECT with b-methyl-p-iodine-123 iodophenyl-pentadecanoid acid (BMIPP) and 99Tc sestamibi and by PET with F-18 2-fluoro 2-deoxyglucose (FDG) or carbon-11 labelled palmitate, demonstrated changes in metabolism of RV in response to various stimuli, in particular in patients with RV pressure overload.^54^

Although nuclear assessment of the RV cannot be considered as the first-choice imaging modality, in selected clinical situations, it can overcome limitations of other techniques (e.g. dependency on geometric assumption for function evaluation of RV as required by other imaging systems) or replace other modalities, when they are not indicated, as in the case of CMR in patients with implantable devices.

## RV assessment by cardiac CT

With the recent improvements in the temporal and spatial resolution of the scanners, cardiac CT can be used to assess RV volume and function,^55^ of particular interest for patients undergoing CT for other clinical indications, such percutaneous intervention of the tricuspid valve or congenital defects or arrhythmias ablation. However, a retrospective ECG-gated acquisition throughout the cardiac cycle is required and ECG-tube current modulation is often implemented to minimize radiation exposure. Previous studies that compared CT measurements of RV volume and function with CMR as the reference standard, showed variable results in terms of agreement,^23,24^ partially due to the difference in RV segmentation methods.

## Conclusions

A systematic and thorough evaluation of RV size, shape, and function provides important diagnostic and prognostic information in most of patients with cardiovascular disease and should be performed by using in a complementary fashion the different imaging modalities available, knowing their strengths and weaknesses. For RV function assessment, echocardiography represents the first line imaging modality in most of patients, and correction of the various measurements for after-load (or the use of less load-dependent approaches) should be attempted, especially when an increase in pulmonary pressures is suspected. CMR, although considered the gold standard for RV dimension and function assessment, is often limited by time, costs, and availability, but should be performed particularly when echocardiography is not conclusive, when detailed anatomical information (such as in congenital heart disease) are necessary and when tissue characterization is required. Cardiac CT is currently used to provide additional RV evaluation in patients undergoing CT for other clinical indications. Finally, nuclear imaging could be performed when information on RV perfusion and metabolism are of importance and the expertise is available.

**Conflict of interest:** Nina Ajmone Marsan received speaker fees from GE Healthcare and Abbott Vascular and has been in the Medical Advisory Board of Philips Ultrasound. Other authors have no conflict of interest to disclose.

Data availability

No new data were generated or analysed in support of this research.
